# Targeting HIF-1 alpha transcriptional activity drives cytotoxic immune effector cells into melanoma and improves combination immunotherapy

**DOI:** 10.1038/s41388-021-01846-x

**Published:** 2021-06-21

**Authors:** Audrey Lequeux, Muhammad Zaeem Noman, Malina Xiao, Kris Van Moer, Meriem Hasmim, Alice Benoit, Manon Bosseler, Elodie Viry, Tsolere Arakelian, Guy Berchem, Salem Chouaib, Bassam Janji

**Affiliations:** 1 Tumor Immunotherapy and Microenvironment (TIME) group, Department of Oncology, Luxembourg Institute of Health (LIH), Luxembourg City, Luxembourg; 2 Department of Hemato-oncology, Centre Hospitalier du Luxembourg, Luxembourg City, Luxembourg; 3 INSERM UMR 1186, Integrative Tumor Immunology and Genetic Oncology, Gustave Roussy, Villejuif, France; 4 Thumbay Research Institute of Precision Medicine, Gulf Medical University, Ajman, United Arab Emirates

**Keywords:** Cancer microenvironment, Cancer immunotherapy, Cancer therapeutic resistance, Melanoma, Tumour immunology

## Abstract

Hypoxia is a key factor responsible for the failure of therapeutic response in most solid tumors and promotes the acquisition of tumor resistance to various antitumor immune effectors. Reshaping the hypoxic immune suppressive tumor microenvironment to improve cancer immunotherapy is still a relevant challenge. We investigated the impact of inhibiting HIF-1α transcriptional activity on cytotoxic immune cell infiltration into B16-F10 melanoma. We showed that tumors expressing a deleted form of HIF-1α displayed increased levels of NK and CD8^+^ effector T cells in the tumor microenvironment, which was associated with high levels of CCL2 and CCL5 chemokines. We showed that combining acriflavine, reported as a pharmacological agent preventing HIF-1α/HIF-1β dimerization, dramatically improved the benefit of cancer immunotherapy based on TRP-2 peptide vaccination and anti-PD-1 blocking antibody. In melanoma patients, we revealed that tumors exhibiting high CCL5 are less hypoxic, and displayed high NK, CD3^+^, CD4^+^ and CD8^+^ T cell markers than those having low CCL5. In addition, melanoma patients with high CCL5 in their tumors survive better than those having low CCL5. This study provides the pre-clinical proof of concept for a novel triple combination strategy including blocking HIF-1α transcription activity along vaccination and PD-1 blocking immunotherapy.

## Introduction

Most solid tumors are hypoxic and characterized by the presence of poorly oxygenated areas (pO_2_ pressure less than 8 mmHg) [1]. Tumor hypoxia results from an imbalance between low oxygen (O_2_) supply, due to an abnormal vascularization, and high O_2_ consumption by tumor cells, which exhibit a marked increase in proliferation and an exacerbated metabolism [2]. Hypoxia-Inducible Factor-1 alpha (HIF-1α) is the major factor mediating the adaptive response to changes in tissue oxygen level [3]. While HIF-1α is rapidly degraded in cells under normoxia conditions, it is stabilized under hypoxia conditions. The translocation of stabilized HIF-1α to the nucleus and its binding with HIF-1β ARNT (Aryl hydrocarbon Receptor Nuclear Translocator) is a key event to activate the expression of several genes involved in various biological processes [4].

Hypoxia contributes to the failure of conventional cancer therapies such as chemotherapy [5] and radiation therapy [6], while data underlining its real impact on immunotherapy efficacy are still missing. Hypoxic stress is a critical microenvironmental factor impairing the antitumor immune response and activating multiple pathways leading to the emergence of resistant tumor cells. We have reported that tumor hypoxia allows tumor cells to escape cytotoxic effector T lymphocytes (CTL) and Natural Killer (NK) mediated killing through the activation of autophagy [7–9]. We have also demonstrated that, through the secretion of microvesicles containing immunosuppressive molecules (i.e., TGF-β, miRNA23a), hypoxic tumor cells are capable of impairing the cytotoxic function of NK cells [10]. In addition, hypoxic stress modulates the composition and function of the immune infiltrate [11–13]. Depending on the nature of the microenvironment, immune cells present in the hypoxic tumor microenvironment may not be able to fulfill their function and could be corrupted to support tumor growth. Hypoxia enhances the immunosuppressive properties of Myeloid-Derived Suppressor Cells (MDSCs) toward T cells by a mechanism involved in the regulation of the immune checkpoint PD-L1 expression on the surface of MDSCs [14–16]. Hypoxia is also involved in the overexpression of other immune checkpoints such as the macrophage immune checkpoint CD47 [17] and the V-domain Ig suppressor of T-cell activation (VISTA) on the surface of MDSCs [18].

Based on the results described above, developing pharmacological agents to modulate HIF-1α signaling pathway has recently inspired significant interest. Indeed, several sub-types of drugs has been described to inhibit HIF-1α activity including inhibitors of HIF-1α/HIF-1β dimerization (e.g., acriflavine) [19, 20]. In this study, we evaluated in vivo the impact of suppressing the transcriptional activity of HIF-1α, by deleting the domain responsible for its dimerization with HIF-1β, on the infiltration of major cytotoxic immune cells into B16-F10 melanoma. The deletion of this particular domain mimics the mode of action of acriflavine (ACF). Our results revealed that suppression of the transcriptional activity of HIF-1α induced the infiltration of NK cells and CD8^+^ T cells in the tumor microenvironment of melanoma. Such infiltration was associated with the release of CCL2 and CCL5 chemokines in the tumor microenvironment. In addition, simultaneous boosting of the CD8^+^ T cell response, using a TRP-peptide vaccination strategy, along with anti-PD-1 and ACF in hypoxic B16-F10 tumors, promoted tumor regression in vivo.

## Results and discussion

### Deleting HIF-1β binding domain in HIF-1α prevents its transcriptional activity in melanoma cells

**Fig. 1:**
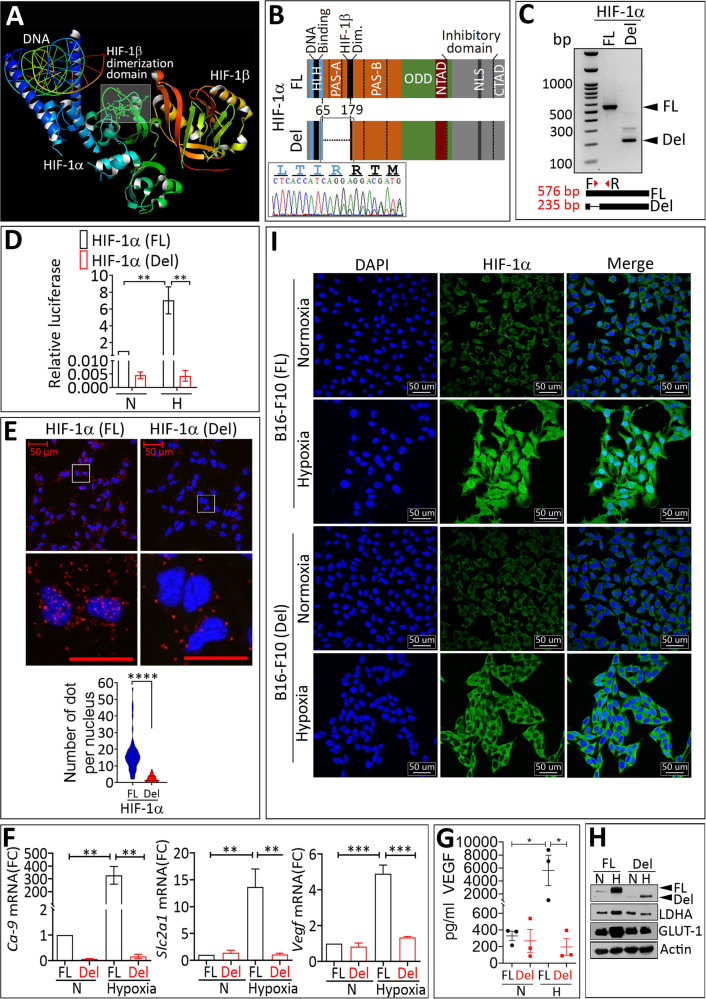
Deletion of the heterodimerization domain of HIF-1α inhibits its transcriptional activity in vitro. **A** Crystal structure of the heterodimeric HIF-1α: ARNT Complex with Hypoxia-response element (HRE) DNA generated from Protein Data Bank Japan (PDBj) (https://pdbj.org/mine_molmil?pdbid = 4zpr). The HIF-1α domain responsible for the dimerization with HIF-1β is outlined. **B** HIF-1α (FL) and HIF-1α (Del) domain structure: The deleted sequence in HIF-1α (Del) goes from amino acid 65 to amino acid 179 within the HIF-1β dimerization domain. HLH, basic helix-loop-helix domain; PAS, PER-ARNT-SIM domains A and B; ODD, oxygen-dependent domain; N- and C-TAD, N- and C-transactivation domains; NLS, nuclear localization signal sequence. **C** RT-PCR detection of HIF-1α (FL) and HIF-1α (Del) showing fragments of 576 and 235 bp for HIF-1α FL and HIF-1α, respectively. The panel inside indicates the design strategy of primers. F: Forward primer and R: Reverse primer. **D** Luciferase report assay performed on B16-F10 cells expressing HIF-1α (FL) and HIF-1α (Del) and cultured under normoxia (N) or hypoxia (H). Results are reported as relative Luciferase activity and normalized to the activity detected in control normoxic B16-F10 cells expressing HIF-1α (FL) as shown in the figure. Data represent the mean of four independent experiments with SEM. Statistically significant difference (indicated by asterisks) are shown (**p < 0.005). **E** Proximity Ligation Assay was performed in B16-F10 cells expressing HIF-1α (FL) or HIF-1α (Del) cultured under hypoxia (0.1% pO_2_, H) for 24 h. The appearance of red dots is indicative of the complex formed between HIF-1α/HIF-1β. Lower images represent a magnification of the delineated area of the upper images. Scale bar: 50 µm. Images are representative of eight acquisitions from HIF-1α (FL) or HIF-1α (Del) expressing cells. Lower graph represents the quantification of red dots present in the nucleus of all cells in the upper images. Results are shown as mean ± SEM (error bars). **** = *p* < 0.0001 determined by two-tailed unpaired Student’s *t* test. **F** RT-qPCR quantification of *Ca-9*, *Slc2a1*, and *Vegf* gene expression in HIF-1α FL (FL) and HIF-1α Del (Del) B16-F10 cells cultured under normoxia (21% pO_2_, N) or hypoxia (0.1% pO_2_, H) conditions for 24 h. Results are reported as fold change (FC) and represent the average of three independent experiments. Error bars indicate mean ± SEM. ** = *p* < 0.01, *** = *p* < 0.001 determined by unpaired two-tailed Student’s *t* test. **G** ELISA quantification of VEGF protein levels in the supernatant of cells described in E. Data are reported in pg/ml and represent three independent experiments. Results are shown as mean ± SEM (error bars). * = *p* < 0.05 determined by one-tailed Student’s *t* test. **H** Western-blot analysis of HIF-1α, LDHA, and Glut−1 protein expression in HIF-1α (FL) and HIF-1α (Del) B16-F10 cells cultured under normoxia (21% pO_2_, N) or hypoxia (0.1% pO_2_, H) conditions for 24 h. Actin was used as a loading control. **I** Confocal microscopy analysis of subcellular localization of HIF-1α (green) in B16-F10 cells expressing either HIF-1α (FL) or HIF-1α (Del) cultured under normoxia (21% pO_2_, N) or hypoxia (0.1% pO_2_, H) conditions for 24 h at the same cell density. Nuclei are stained with DAPI (blue). Individual and merged images of HIF-1α and DAPI staining are shown. Scale bar: 50 μm.

We investigated the impact of deleting the HIF-1α domain responsible for HIF-1α dimerization with HIF-1β/ARNT (Aryl hydrocarbon Receptor Nuclear Translocator) (Fig. 1A) on B16-F10 melanoma growth. We used CRISPR/Cas9 technology to cleave specifically the HIF-1α/ARNT dimerization domain, located between the basic Helix-Loop-Helix (bHLH) and PER-ARNT-SIM (PAS) domains on the HIF-1α subunit. DNA sequencing showed a deletion of a 114 amino acid fragment close to the HIF-1α N-terminus. This fragment contains a substantial part of the HIF-1α heterodimerization domain (deletion of 10 amino acids over 22 constituting the HIF-1α heterodimerization domain). Other domains located outside the deleted fragment, such as the DNA-binding domain, located in the bHLH, and the oxygen-dependent degradation domain (ODD), remained intact. The truncated and full-length HIF-1α proteins are therefore termed HIF-1α (Del) and HIF-1α (FL), respectively (Fig. 1B, C). The transcriptional activity of HIF-1α (Del) was abolished, as demonstrated by luciferase activity assay and (Fig. 1D), and the interaction of HIF-1α (Del) with HIF-1β was substantially disrupted as showed by using Proximal Ligation Assay (Fig. 1E). Accordingly, no increase was observed in the expression of HIF-1α downstream target genes such as carbonic anhydrase 9 (*Ca-9*), glucose transporter-1 (*Slc2a1*) and vascular endothelial growth factor (*Vegf*) in cells expressing HIF-1α (Del) under hypoxia (Figure F-G). HIF-1α (Del) protein having smaller molecular weight (Fig. 1H) was still accumulated in hypoxic B16-F10 cells but only in the cytoplasm as demonstrated in Fig. 1I.

### Impairing the transcriptional activity of HIF-1α in melanoma inhibits the tumor growth by driving major cytotoxic immune cells into the tumor microenvironment

**Fig. 2:**
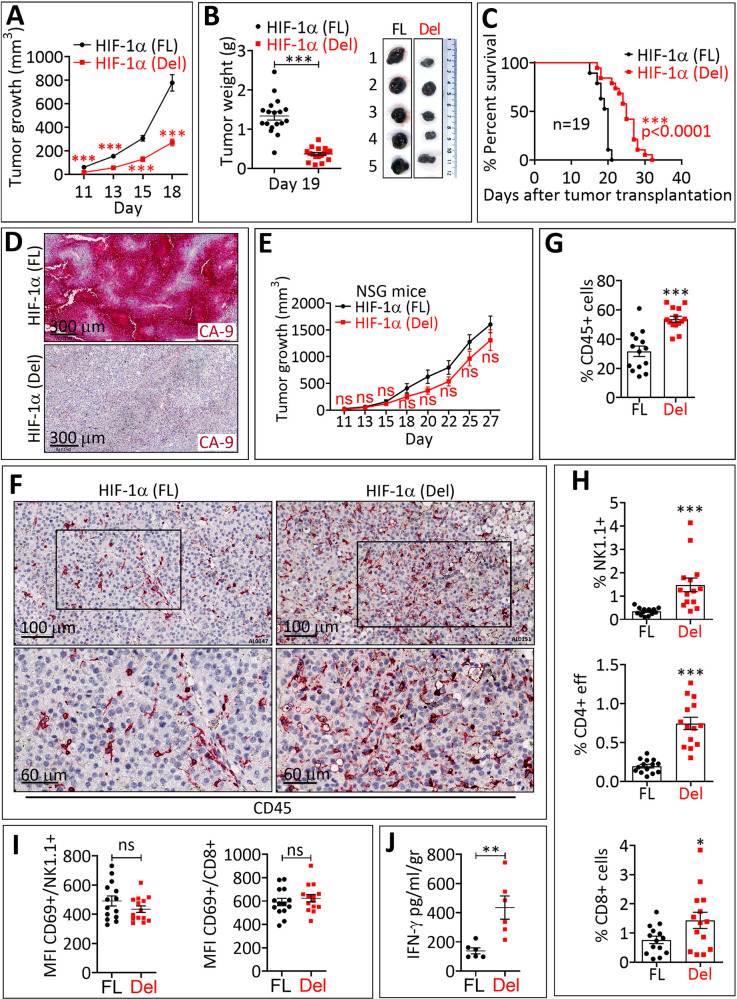
Targeting the transcriptional activity of HIF-1α inhibits B16-F10 tumor growth and weight, improves mice survival, and increases NK and CD8^+^ immune effector cells infiltration. **A** Tumor growth curves of HIF-1α (FL) and HIF-1α (Del) B16-F10 melanoma cells subcutaneously implanted in syngeneic host C57BL/6 mice. Growth curves are reported as the average of 30 (HIF-1α FL) and 29 (HIF-1α Del) mice per group pooled from three independent experiments conducted with at least 6 mice per group. The curve represents mean ± SEM. *** = *p* < 0.001 determined by unpaired two-tailed Student’s *t* test. **B** Weight in grams (g) of HIF-1α (FL) and HIF-1α (Del) B16-F10 melanoma tumors at day 19. Data are reported as the average of 18 (HIF-1α (FL)) and 17 (HIF-1α (Del)) mice pooled from two independent experiments performed with at least 5 mice per group. Results are presented as mean ± SEM. *** = *p* < 0.001 determined by unpaired two-tailed Student’s *t* test. Representative pictures of five HIF-1α (FL) and five HIF-1α (Del) tumors are shown in the right. **C** Kaplan–Meier survival curves of HIF-1α (FL) and HIF-1α (Del) B16-F10 melanoma. Results are generated from 19 tumor-bearing mice per group. Death, tumor necrosis, or tumor size ≥2000 mm^3^ define the occurrence of a death event. *** = *p* < 0.0001 determined by the Log-rank (Mantel-Cox) test. **D** CA-9 immunohistochemical staining (red) performed on HIF-1α (FL) and HIF-1α (Del) B16-F10 melanoma tumors. Scale bar: 300 μm. **E** Tumor growth curves of HIF-1α (FL) and HIF-1α (Del) B16-F10 melanoma cells subcutaneously transplanted in immuno-deficient NOD scid gamma mouse (NSG) mice. Results are reported as an average of 10 (HIF-1α (FL)) and 9 (HIF-1α (Del)) mice per group. Error bars represent mean ± SEM. ns = not significant determined by unpaired two-tailed Student’s *t* test. **F** Immunohistochemical staining of CD45+ cells (red) performed on HIF-1α (FL) and HIF-1α (Del) B16-F10 melanoma. Lower panels represent a magnification of the delineated tumors area of the upper panels. Scale bars: 100 μm (upper panels); 60 μm (lower panels). **G** Quantification of CD45^+^ leukocyte infiltration (gated in live cells) in HIF-1α (FL) and HIF-1α (Del) B16-F10 melanoma tumors. Results are reported as the average of 14 mice per group, pooled from two independent experiments conducted with at least 5 mice per group (each dot represents one tumor). Error bars represent mean ± SEM. *** = *p* < 0.001 determined by two-tailed Student’s *t* test. **H** Quantification of NK cells (NK1.1^+^), CD4^+^ T effector cells (CD4^+^), and CD8^+^ T cells (CD8^+^) percentage infiltrating HIF-1α (FL) and HIF-1α (Del) B16-F10 melanoma by flow cytometry (gated in live CD45^+^ cells). Bars represent the average of 14 mice per group pooled from two independent experiments conducted with at least 5 mice per group (each dot represents one tumor). Error bars represent mean ± SEM. ns = not significant, * = *p* < 0.05 and *** = *p* < 0.001 determined unpaired two-tailed Student’s *t* test. **I** Quantification of CD69^+^ activated NK cells (left panel) and CD8^+^ T cells (right panel) infiltrating the B16-F10 melanoma expressing FL or Del HIF-1α. Data are reported as mean florescence intensity (MFI) and represent the average ± SEM (error bars) of 14 mice per group (each dot represents one tumor). Statistically significant differences (indicated by asterisks) are calculated compared to FL tumors using an unpaired two-tailed Student’s *t* test (ns = not significant). **J** ELISA quantification of IFN-γ secreted in the microenvironment of B16-F10 melanoma expressing HIF-1α (FL) and HIF-1α (Del). Data are reported in pg/ml standardized to excised tumor weight (gr) and represent the mean of 6 tumors per group ± SEM (each dot represents one mouse). Statistically significant differences (indicated by asterisks) are calculated compared to FL tumors using an unpaired two-tailed Student’s *t* test (** = *p* < 0.005).

We next evaluated the impact of deleting the HIF-1α-ARNT dimerization domain on the in vivo tumor growth of B16-F10 melanoma cells. In vitro, there was no significant difference between the proliferation of HIF-1α (Del) and HIF-1α (FL) B16-F10 cells (Supplementary Fig. 1A). We observed a significant inhibition in the growth (Fig. 2A) and weight (Fig. 2B) associated with an improvement in the survival (Fig. 2C) of mice bearing HIF-1α (Del) compared to those bearing HIF-1α (FL). Immunohistochemical (IHC) staining performed on tumor sections at day 19 showed no CA-9 staining in HIF-1α (Del) compared to HIF-1α (FL) tumors, indicating that the transcriptional activity of HIF-1α (Del) protein remains absent in tumors until at least 19 days (Fig. 2D).

ACF is an FDA-approved small molecule inhibiting the dimerization of HIF-1α with ARNT. ACF pretreatment in mice bearing prostate cancer xenografts decreased tumor growth and inhibited vascularization [21]. Digoxin and other cardiac glycosides that inhibit HIF-1α protein synthesis blocked tumor growth [22]. The above-mentioned studies used immunodeficient mice to investigate the impact of targeting HIF-1α on tumor growth. In this study, our main objective was to use immunocompetent mice to study the impact of targeting HIF-1α on immune cell infiltrate within the tumor bed. Of note, no difference in the growth of HIF-1α (Del)-tumors and HIF-1α (FL)-tumors was observed in immunocompromised NOD scid gamma (NSG) mice lacking mature B, T, and NK cells (Fig. 2E), highlighting the involvement of the host immune system in inhibiting HIF-1α (Del) tumor growth in mice. We next assessed the infiltration of CD45^+^ immune cells and major cytotoxic immune cells into HIF-1α (FL)- and HIF-1α (Del)- tumors. Figure 2F–H shows a significant increase in the infiltration of live CD45^+^, NK, CD4^+^, and CD8^+^ T cells into HIF-1α (Del) tumors as compared to HIF-1α (FL) tumors. By analyzing the expression of the early activation marker CD69 on NK and CD8 T cells and assessing the level of IFN-γ in the tumor microenvironment [23], we showed no significant difference in the expression of CD69 in NK and CD8^+^ T cells (Fig. 2I), but a significant increase in IFN-γ in HIF-1α (Del) compared to HIF-1α (FL) B16-F10 tumors (Fig. 2J). Furthermore, Supplementary Fig. 1B showed a significant increase in the infiltration of Foxp3^+^ Treg cells in HIF-1α Del compared to HIF-1α FL tumors, which exhibited a significant decrease in the expression of the activation marker CD69, but not the exhaustion marker PD-1. Nevertheless, our data suggest that the increased infiltration of CD4^+^ T cells, observed in HIF-1α-deleted tumors could be partially related to an increased infiltration of Treg cells.

**Fig. 3:**
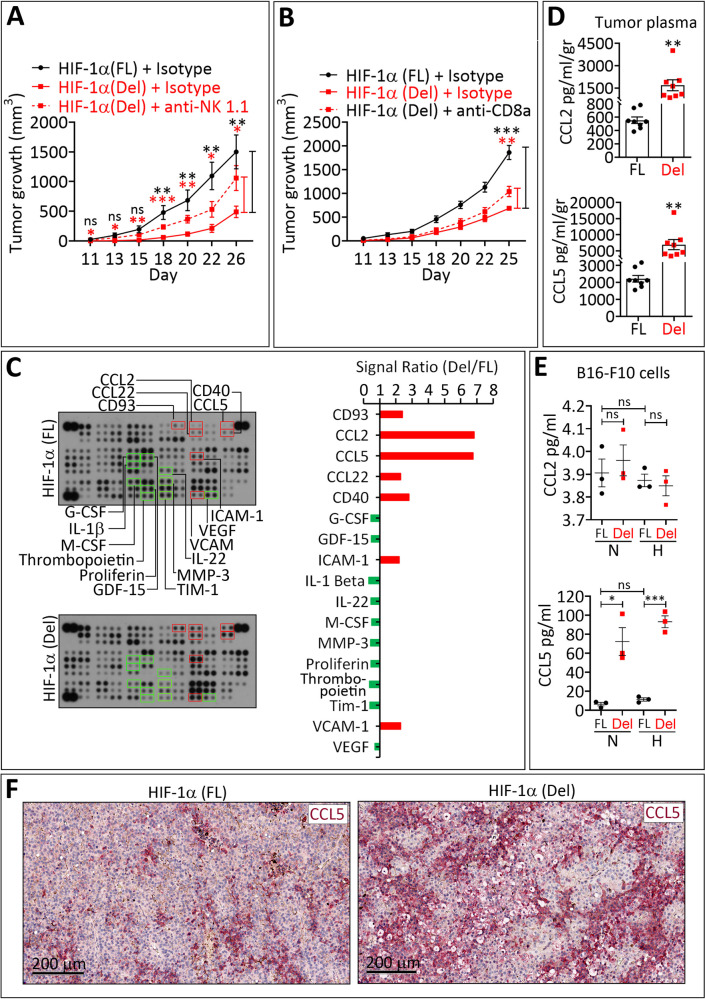
The infiltration of NK and CD8^+^ T cells into HIF-1α Del tumors is associated with the release of CCL5 from tumor cells. **A** Tumor growth curves of HIF-1α (FL) and HIF-1α (Del) B16-F10 cells transplanted in C57BL/6 mice untreated (Isotype) or treated with NK-depleting antibody (anti-NK1.1). Mice were injected intraperitoneally with 200 µg of Isotype or anti-NK1.1 three days before the engraftment tumor cells and at days 0, 4, 8, 11, 15, 18 and 22 after the engraftment. Results are reported as average of 10 mice per group for [HIF-1α (FL) + Isotype] and [HIF-1α (Del) + anti-NK] groups, and 8 mice per group for [HIF-1α (Del) + Isotype] group. Error bars represent mean ± SEM. ns = not significant, * = *p* < 0.05, ** = *p* < 0.01 and *** = *p* < 0.001 determined by unpaired two tailed Student’s *t* test. **B** Tumor growth curves of HIF-1α (FL) and HIF-1α (Del) B16-F10 cells transplanted in untreated (Isotype) or CD8a-depleted (anti-CD8a) C57BL/6 mice. Mice were injected intraperitoneally with 200 µg of Isotype or anti-CD8a antibody three days before the engraftment tumor cells and at days 0, 4, 8, 11, 15, 18 and 22 after the engraftment. Data are reported as average of 8 mice per group for [HIF-1α (FL) + Isotype] group, 5 mice per group for [HIF-1α (Del)+ Isotype] and 7 mice per group for [HIF-1α (Del)+ anti-CD8]. Error bars represent mean ± SEM. ns = not significant, ** = *p* < 0.01 and *** = *p* < 0.001 determined by unpaired one tailed Student’s *t* test. **C** Cytokines/chemokines profiling in tumor lysate of HIF-1α (FL) and HIF-1α (Del)B16-F10 tumors using Mouse XL Cytokine Array. Left panel: Cytokines/chemokines up- and down-regulated in HIF-1α (Del) compared to HIF-1α (FL) B16-F10 tumor are highlighted in red and in green, respectively. Right panel: signal ratio of HIF-1α Del/HIF-1α FL (Del/FL). Mouse XL Cytokine Array for cytokines/chemokines profiling was performed once. **D** ELISA quantification of CCL2 and CCL5 in tumor plasma of HIF-1α (FL)and HIF-1α (Del)B16-F10 tumors. Data are reported in pg/ml/g of tumor. Bars represent an average of 8 mice. Error bars represent mean ± SEM. ** = *p* < 0.01 determined by unpaired two tailed Student’s *t* test. **E** ELISA quantification of CCL2 and CCL5 released in the supernatant of HIF-1α (FL) and HIF-1α (Del) B16-F10 cells cultured under normoxia (21% pO_2_, N) or hypoxia (0.1% pO_2_, H) conditions for 24 h. Bars are mean ± SEM from three independent experiments. ns = not significant, *** = *p* < 0.001 determined by unpaired two tailed Student’s *t* test. **F** Immunohistochemical staining of CCL5 (red) performed on HIF-1α (FL)and HIF-1α (Del) B16-F10 tumors. Scale bars: 200 μm.

The decrease in HIF-1α (Del) tumor growth was no longer observed when host NK cells were depleted from tumor-bearing mice (Fig. 3A and Supplementary Fig. 1C). CD8^+^ T cell depletion also partially rescued the growth inhibition of HIF-1α (Del) tumors but to a lesser extent (Fig. 3B and Supplementary Fig. 1D). These results highlight the major contribution of NK cells, but they do not exclude the role of CD8^+^ T cells, in the inhibition of HIF-1α (Del) tumor growth.

### Inhibiting the transcriptional activity of HIF-1α enhances the release of CCL5 in the tumor microenvironment

To gain insight into the cytokine/chemokine network involved in driving immune cells into HIF-1α (Del) tumors, we used large-scale Proteome Profiler Mouse XL Cytokine Array combined to ELISA assay. We identified CCL2 and CCL5 as the major chemokines secreted in HIF-1α (Del) tumors as compared to HIF-1α (FL) tumors (Fig. 3C, D). Supplementary Figure 2A showed a significant increase in the mRNA of CCL5, but not CCL2, in HIF-1α (Del) compared to HIF-1α (FL) tumors.

An ELISA assay of CCL2 in the extracellular medium of HIF-1α (FL) and HIF-1α (Del) cells cultured in vitro showed no regulation of CCL2 by hypoxia even when HIF-1α was truncated. CCL5 extracellular levels were also not regulated by hypoxia but HIF-1α truncation under both normoxia and hypoxic led to a consistent increase in CCL5 levels (Fig. 3E). *Ccl2* and *Ccl5* mRNA expression in HIF-1α (FL) and HIF-1α (Del) cells provided similar results, but no significant increase in *CCL5* mRNA was observed in HIF-1α (Del) compared to HIF-1α (FL) cells under normoxia (Supplementary Fig. 2B).

Our key results reported in B16-F10 cells related to the increase of CCL5 can be reproduced in 4T1 breast cancer cells, as the depletion of HIF-1α (evidenced by the failure of hypoxia to induce *Ca-9* and *Slc2a* mRNA and protein) had no impact cell proliferation in vitro, but significantly induced the release of CCL2 and CCL5 under both normoxia and hypoxia (Supplementary Fig. 2C-F).

Nevertheless, in B16-F10 cells, our data indicate that CCL5 overexpression detected in tumors expressing HIF-1α (Del) is most likely released by B16-F10 tumor cells while CCL2 is probably released by the microenvironment of HIF-1α (Del) tumors. CCL5 overexpression was further confirmed by IHC on tumor sections (Fig. 3F). Further experiments must be carried out to determine which cell subtypes are responsible for the release of CCL2 in the microenvironment of HIF-1α (Del) tumors. We believe that, relative to CCL2, CCL5 regulation in B16-F10 tumor cells following HIF-1α deletion plays a major chemotaxis role in driving immune effectors and subsequently reshaping the tumor microenvironment. Therefore, it would be interesting to analyze the infiltration of immune cells reported to be a major source of CCL2 such as MDSC and tumor associated macrophages (TAMs).

Depending on its expression level, CCL2 can play a role as pro- or anti-tumorigenic factor [24]. In B16-F10 melanoma, it has been reported that targeting CCL2 reduced malignant pleural effusion and enhanced mice survival compared to control [25]. Conversely, other study showed that CCL2 inhibits B16-F10 tumor growth in C57Bl/6 mice by inducing the homing of adoptively transferred human cytotoxic lymphocytes (CTLs) to the tumor microenvironment [26]. Therefore, we believe that the role of CCL2 and the mechanism involved in its indirect regulation following HIF-1α deletion need to be more extensively investigated. Our attempt to stain CCL2 in the tumor microenvironment of HIF-1α (Del) tumors failed (data not shown). This failure is most likely due to the relatively weak expression of CCL2 compared to CCL5.

### Treating melanoma-bearing mice with ACF increases the release of CCL5 in the tumor microenvironment and improves the infiltration of CD3+ and CD4+ effector cells

**Fig. 4:**
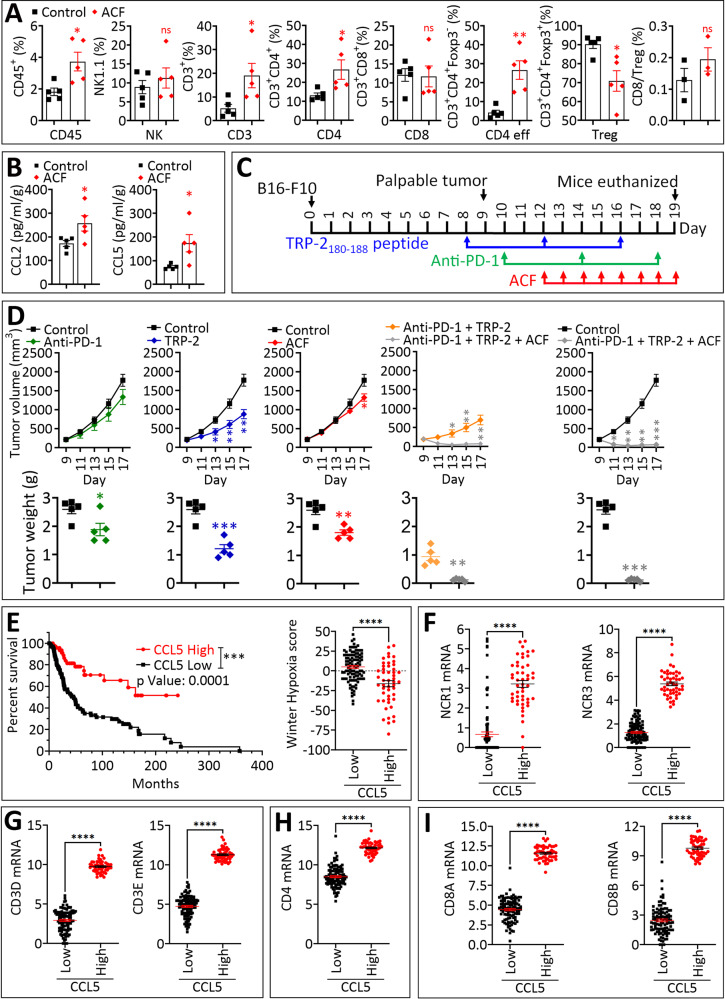
Pharmacological targeting of HIF-1α transcription activity using Acriflavine improves the therapeutic benefit of anti-PD-1 immune checkpoint- and TRP-2 vaccine-based cancer immunotherapy. **A** Quantification of the infiltration of CD45^+^ cells and lymphoid cells (NK, CD3^+^, CD4^+^, CD4^+^ eff, CD8, and Treg) and CD8/Treg ratio in control and ACF-treated B16-F10 tumors. Data are reported as the percentage (%) of CD45 and represent the mean ± SEM (error bars) of 5 tumors per group (each dot represents one tumor). Statistically significant differences (indicated by asterisks) are calculated compared to control tumors using an unpaired two-tailed Student’s *t* test (ns = not significant; * = *p* < 0.05; ** = *p* < 0.01). **B** ELISA quantification of CCL2 and CCL5 in tumor plasma of control and ACF-treated tumors. Data are reported in pg/ml/g of tumor and represent the mean ± SEM (error bars) of 5 tumors (each dot represents one tumor). Statistically significant differences (indicated by asterisks) are calculated compared to control tumors using an unpaired two-tailed Student’s *t* test (* = *p* < 0.05). **C** Schematic representation of the triple combination strategy including anti-PD-1, ACF, and TRP-2_180-188_ peptide in B16-F10 tumor-bearing mice. 0.2 × 10^6^ B16-F10 cells were subcutaneously (s.c.) injected into the right flank of syngeneic host C57BL/6 mice at day 0. When tumors were palpable (typically at day 8), TRP-2_180-188_ peptide was injected s.c. (100 μg TRP-2_180-188_ and 50 μg CpG ODN emulsified in 100 μL of IFA) in the left posterior part of mice. The vaccination was boosted twice at day 12 and day 16 (blue arrows). Mice were injected intraperitoneally with 100 µg of anti-PD-1 or control isotype at days 10, 14, and 18 (green arrows). Daily ACF treatment (2 mg/kg) was administered intraperitoneally from day 12 to 19 (red arrows). **D** B16-F10 tumor growth curves and tumor weight of mice treated with either isotype (control), combined with anti-PD-1, TRP-2_180-188_, or ACF. Upper panels: B16-F10 tumor growth curves in mice treated with mono, double or triple combination of anti-PD-1, TRP-2_180-188_, and ACF. Lower panels: B16-F10 tumor weight (grams, g) at day 19. Results are reported as the average of 5 mice per group and shown as mean ± SEM (error bars). Absence of asterisks indicates no significant difference, * = *p* < 0.05, ** = *p* < 0.01, and *** = *p* < 0.001 determined by unpaired two-tailed Student’s *t* test. **E** Left panel: Kaplan–Meier median survival curves of the two melanoma patient cohorts expressing high and low CCL5 mRNA. Patients displaying high CCL5 have significantly improved median survival compared to those having low CCL5. A *p* value of 0.0001 was determined using the Log-rank (Mantel-Cox) test. Right panel: Winter hypoxia score of patients expressing low and high CCL5 mRNA. **F-I** The expression of markers for different immune cells in melanoma patient cohorts described in (**E**). (**F**) NCR1 and NCR3 for NK cells; (**G**) CD3D and CD3E for CD3 cells; (**H**) CD4 for CD4; and (**I**) CD8A and CD8B for CD8 T cells. Statistically significant differences (indicated by asterisks) are calculated compared to patients expressing low CCL5 mRNA using an unpaired two-tailed Student’s *t* test (****** = *p* < 0.0001).

Our results described above strongly argue that therapeutic strategies preventing HIF-1α/HIF-1β dimerization would improve cancer immunotherapies by inducing the infiltration of immune effectors. We therefore evaluate the role of ACF on the infiltration of lymphoid cells into B16-F10 melanoma. We first showed that ACF treatment of cells, pre-cultured in vitro under hypoxia, significantly decreased hypoxia-dependent overexpression of *Slc2a1* and *Vegf* in a dose-dependent manner. Under these experimental conditions in vitro, we did not observe any changes in the mRNA expression of Ccl2 and Ccl5 (Supplementary Fig. 2G). This could be related to the experimental conditions of the in vitro treatment with ACF or/and to the mode of action of ACF which could be broader than a strict inhibition of HIF-1 α/β heterodimerization. However, treatment of B16-F10 tumor bearing mice with ACF (2 mg/Kg) significantly increased the infiltration of CD45^+^ cells as well as CD3^+^, total CD4^+^ and CD4^+^ effector T cells associated with a decrease in the infiltration of Treg cells (Fig. 4A). The activation marker CD69 and the exhaustion marker PD-1 were up- and down-regulated, respectively, only in CD4^+^ effector T cells (Supplementary Fig. 3A–C). Interestingly, ACF-treated tumors showed a significant increase in both CCL2 and CCL5 in the tumor microenvironment (Fig. 4B).

### Combining ACF improves the therapeutic benefit of TRP-2-based vaccine and anti-PD-1 in melanoma

We next evaluated the therapeutic benefit of combining ACF with two well-known immunotherapy approaches (anti-PD-1 immune checkpoint blockade antibody and TRP-2_180-188_ peptide vaccination strategy) (Fig. 4C) which reactivate CD8^+^ T cells in B16-F10 melanoma [9, 27]. Our results showed that anti-PD-1 treatment alone had no impact on B16-F10 tumor growth and tumor weight. Although the TRP-2-based vaccine resulted in a significant inhibition of tumor growth, tumors continued to grow at a slower but constant rate. ACF treatment alone induced a moderate inhibition of tumor growth and tumor weight in B16-F10 melanoma (Fig. 4D and Supplementary Fig. 3D). Interestingly, triple combination therapy (anti-PD-1 + TRP-2 + ACF) completely inhibited tumor growth compared to either the double combination therapy (anti-PD-1 + TRP-2) or control (Fig. 4D), indicating that ACF can improve cancer immunotherapy approaches based on immune checkpoint blockade and peptide-based vaccination strategies. Our data are in line with several studies highlighted the relevance of targeting hypoxia-associated pathways in tumors to enhance immunotherapy efficacy [28, 29].

### Assessing the therapeutic value of CCL5 in melanoma patients

We next investigated whether infiltration of major cytotoxic immune cells correlates with the hypoxia status in 473 melanoma patients from TCGA database. We identified two groups of melanoma expressing low and high level of CCL5 mRNA and showed that the median survival of melanoma patients expressing high CCL5 is significantly better than those expressing low CCL5 (Fig. 4E). Interestingly, according to Winter hypoxia score, “CCL5 high melanomas” are less hypoxic than “CCL5 low melanomas” (Fig. 4E, right panel). Remarkably, the infiltration of NK cells, CD3, CD4, and CD8 T cells, assessed by the mRNA expression of their respective markers NCR1 and NCR2, CD3D and CD3E, CD4, and CD8A and CD8B, is increased in “CCL5 high melanomas” compared to “CCL5 low melanomas” (Fig. 4F–I). Data about TCGA patients are provided in Supplementary Table 1. It should highlighted that hypoxic signature was found enriched in metastatic melanoma patients non-responding to anti-PD-1 [30].

## Concluding remarks

Our study revealed that blocking the transcriptional activity of HIF-1α, by disrupting HIF-1α/HIF-1β dimerization, significantly inhibited B16-F10 melanoma growth and increased the infiltration of NK and CD8^+^ T cells into the tumor microenvironment by a mechanism involving the release of CCL2 and CCL5 chemokines. Together, our data strongly argue that therapeutic strategies disrupting HIF-1α/HIF-1β dimerization would be able to switch the tumor microenvironment from immunosuppressive to immunopermissive for the infiltration of NK and CD8^+^ effector T cells. Such strategies could be used to improve cancer vaccination and immune checkpoint blockade-based cancer immunotherapies in non-responder melanoma patients.

## Materials and methods

Because of the word limits, materials and methods can be found in the Supplementary Information.

## Supplementary information

Supp Materials

Legends for Supp Figures

Supp Fig 1

Supp Fig 2

Supp Fig 3

Table 1
