# Supplementary material for: Targeting HIF-1 alpha transcriptional activity drives cytotoxic immune effector cells into melanoma and improves combination immunotherapy
Source: Oncogene. 2021 Jun 21;40(28):4725–35. doi: 10.1038/s41388-021-01846-x (PMC8282500; doi:10.1038/s41388-021-01846-x)
Supplement: Supplementary file 1 — Supp Materials [file 41388_2021_1846_MOESM1_ESM.docx]

**Supplementary Materials and Methods**

**Cell culture, transfection and XTT assay**

B16-F10 and 4T1 cell lines were obtained from ATCC and cultured as previously described (1). CRISPR/Cas9 and HIF-1α CRISPR/Cas9 plasmids were obtained from Santa Cruz Biotechnology and transfected into cells according to the manufacturer’s protocol. The hypoxic condition was performed using a hypoxia chamber (RUSKINN I-CONIC) at 0.1% pO_2_ for 24 hours. Acriflavine hydrochloride (Sigma Aldrich) treatment was carried out for the last 6h of hypoxia culture at the following concentrations: 5, 10, 20, and 30 µM. The doubling time was measured using a colorimetric cell viability assay (XTT; Sigma Aldrich). Equal amount of cells was plated in 8 replicates in 96 wells with 100ul of complete medium. The doubling time was determined at 24-48, 48-72 and 24-72 hours after adding of 50ul XTT (1mg/ml) solution in each well by measuring the absorbance at 450 and 630nm.

**RNA extraction and real time quantitative qPCR**

Total RNA was extracted and RT-qPCR was performed as previously described (1). Primer sequences are available upon request. RNA for sequencing was reverse-transcribed and amplified by PCR. PCR products were extracted on agarose gel and purified using a NucleospinRNA Plus (Macherey-Nagel) kit. Sequencing was performed by the LGC Genomics Company using the following primers: forward 5’-TGA GTT CTG AAC GTC GAA AAG A-3’ and reverse 5’-AAT ATG GCC CGT GCA GTG AA-3’.

**Flow cytometry**

Tumors were dissociated in DMEM complete medium and processed as previously described (1). The following fluorochrome-conjugated antibodies were used: anti-CD45, anti-CD3ɛ, anti-CD25 (all purchased from BD Horizon); anti-FoxP3 and anti-CD4 (all purchased from eBioscience); anti-mouse CD69, anti-NK1.1, anti-mouse CD279 (PD-1) and anti-CD8a (all purchased from Biolegend). The LIVE/DEAD® Fixable Near-IR Dead Cell Stain Kit (Thermo Fisher Scientific) was used as a viability dye. For intracellular staining of Foxp3/Transcription Factor, a fixation/permeabilization kit from eBioscience was used according to the manufacturer’s recommended protocol.

**Elisa and Mouse cytokine profiling**

Tumor plasmas were generated from dissociated tumors in DMEM medium. After centrifugation, supernatants were collected and concentrated using Protein Concentrator PES, 3K MWCO (88526, Thermo Fisher Scientific). For in vitro experiments, equal amounts of cells were seeded in 6 well dishes. Twenty-four hours later, cells were serum-deprived and cultured under hypoxia at 0.1% pO_2_ for 24 hours. CCL2, CCL5, IFN-γ and VEGF present in the supernatant were quantified using mouse CCL2/JE/MCP-1 DuoSet Elisa, CCL5/RANTES DuoSet Elisa, IFN-gamma DuoSet Elisa and VEGF DuoSet Elisa, which were purchased from R&D Systems. Proteome Profiler Mouse XL Cytokine Array (R&D Systems) was used for profiling different cytokines and chemokines using equal amounts (µg) of tumor lysates.

**Immunohistochemistry and immunofluorescence**

Mice harvested tumors were fixed in 4% paraformaldehyde and 70% ethanol and embedded in paraffin. CD45, CA-9, and CCL5 staining was performed by HistoWiz Company (NY, USA). For immunofluorescence, cells were seeded at the same density for all experimental conditions, fixed with paraformaldehyde 4% and then permeabilized with 0.25% Tween20 before being stained with HIF-1α (Novus Biologicals) and DAPI and incubated with appropriate fluorescently conjugated secondary antibody (Thermo Fisher Scientific).

**Depletion of NK and CD8**

NK and CD8 depleting antibodies were administred to mice three day prior the engraftment of tumor cells. Blood samples were collected from the eye of mice. Blood cells were stained with the following fluorochrome-conjugated antibodies for 30 minutes: PE anti-NK1.1, APC anti-CD3, FITC anti-CD3 and APC anti-CD8. RBC lysis buffer 10x was used to lyse red blood cells.

***In vivo* blocking antibodies and TRP2 vaccination**

The following antibodies were used for in vivo blocking/depletion: InVivoMab anti-mouse NK1.1 (BE0086), InVivoMab mouse IgG2a isotype control (BE0085), InVivoMab anti-mouse CD8 alpha (BE0061), InVivoMab rat IgG2b isotype control (BE0090), InVivoMab anti-mouse PD-1 (CD279) (BE0273), and InVivoMab rat IgG2a isotype control (BE0089), which were purchased from BioXCell, diluted in InVivoPure pH 7.0 Dilution Buffer (IP0070), and administered as indicated in the corresponding figures. Lyophilized TRP-2_180-188_ peptide (SVYDFFVWL; Proteogenix) was diluted in dimethyl sulfoxide. Synthetic CpG ODN 1826 (TCCATGACGTTCCTGACGTT; Oligofactory) was dissolved in PBS.

**Luciferase reporter assay**

B16-F10 cells expressing HIF-1α (FL) and HIF-1α (Del) were plated in two 48-well plates (10^4^ cells/well) and transfected the next day with a Hypoxia Responsive Element (HRE)-luciferase reporter construct using Fugene transfection reagent (#E2311; Promega) according to the manufacturer’s instructions. HRE-luciferase construct was kindly provided from Navdeep Chandel (Addgene plasmid # 26731) (http://n2t.net/addgene:26731; RRID:Addgene_26731) (2). 24h post transfection, one plate was transferred to hypoxia incubator (0.1% pO2) while the second plate was kept in normoxic conditions. 24h later, hypoxic and normoxic cells were lysed in luciferase reagent (#E6120: Promega) and luminescence was measured in a luminometer.

**Proximity Ligation Assay (PLA)**

Proximity Ligation Assay (PLA) allows evaluating *in situ* protein-protein interactions with high specificity and sensitivity. We used this technology to assess the interaction between HIF-1α (FL) or HIF-1α (Del) with HIF-1β. B16-F10 cells expressing HIF-1α (FL) or HIF-1α (Del) were seeded on μ-Slide VI 0.4 (Ibidi) and cultured under hypoxia (0.1% pO_2_, H) condition for 24 hours. Cells were fixed for 15 min with 4% PFA in the hypoxia chamber and permeabilized with 0.25% Tween-20 at RT. The PLA was performed according to the manufacturer’s recommendations (Duolink PLA Fluoresence Protocole, Sigma-Aldrich). Z-stacking images were acquired on confocal LSM880 Airy (Carl Zeiss) and scale bars were added using ZEN software. The following primary antibodies were used HIF-1α (Novus, NB100-449) and HIF-1β (Novus, NB100-124). The appearance of red dots is indicative of the complex formed between HIF-1α/HIF-1β and the red dots were quantified by Image J software.

**Western blotting**

Cells and tumors were lysed in RIPA lysis buffer (20-188; Millipore) and processed as previously described (1). Anti-HIF-1α, - LDHA, and -GLUT-1 antibodies were from Cell signaling, and anti-actin antibody was from Sigma.

**Mice and *in vivo* study approval**

HIF-1α (FL) and HIF-1α (Del) B16-F10 cells (2.3 x 10^5^ cells per mouse) were injected subcutaneously in seven-week-old C57BL/6 (Janvier Labs) or immunodeficient NOD scid gamma (NSG) mice. Tumor volumes were measured as previously described (1). Mice were handled according to European Union guidelines and experimentation protocols were approved by the LIH ethical committee, Animal Welfare Society Luxembourg (agreements n. LECR-2017-02 and LECR-2018-12).

**Melanoma patient data mining**

TCGA skin cutaneous melanoma (SKCM) patient cohort was downloaded from cBioPortal (<http://www.cbioportal.org/>). Patients ID displaying high and low CCL5 expression were extracted. The vital status and median survival of each patient as well as Winter hypoxia score were downloaded from the TCGA database. The mRNA expression level (Batch normalized from Illumina HiSeq_RNASeqV2) (log2) of markers for NK (NCR1 and NCR3), CD3 (CD3D and CD3E), CD4 and CD8 (CD8A and CD8B) in patients displaying high and low CCL5 was identified. The differential expression of genes of interest was done using GraphPad software. The median survival and the *P*-value were calculated by using the Log-rank (Mantel-Cox) test in GraphPad software.

**Statistical analysis**

Statistical analyses were performed using GraphPad Prism 8. An unpaired two- or one-tailed t-test was used to determine *P*-value between indicated groups. Results were represented as mean ± SEM (standard error of the mean). A *P*-value of <0.05 matches for statistical significance * P<0.05; ** P<0.01; *** P<0.001; ns: not significant.

**References**

1. Noman MZ, Parpal S, Van Moer K, Xiao M, Yu Y, Viklund J, et al. Inhibition of Vps34 reprograms cold into hot inflamed tumors and improves anti-PD-1/PD-L1 immunotherapy. Sci Adv. 2020;6(18):eaax7881.

2. Emerling BM, Weinberg F, Liu JL, Mak TW, Chandel NS. PTEN regulates p300-dependent hypoxia-inducible factor 1 transcriptional activity through Forkhead transcription factor 3a (FOXO3a). Proc Natl Acad Sci U S A. 2008;105(7):2622-7.
