# Supplementary material for: Targeting HIF-1 alpha transcriptional activity drives cytotoxic immune effector cells into melanoma and improves combination immunotherapy
Source: Oncogene. 2021 Jun 21;40(28):4725–35. doi: 10.1038/s41388-021-01846-x (PMC8282500; doi:10.1038/s41388-021-01846-x)
Supplement: Supplementary file 2 — Legends for Supp Figures [file 41388_2021_1846_MOESM2_ESM.docx]

**Legends for supplementary Figure 1**

**(A)** XTT assay to evaluate the doubling time of HIF-1α (FL) and HIF-1α (Del) B16-F10 cells at indicated time intervals under hypoxia (0.1% p0_2_) conditions. The average of eight replicates is reported. The error bars represent mean ± SEM. ns = not significant determined by an unpaired two-tailed Student’s *t*-test.

**(B)** Quantification of the infiltration of Treg (left panel), PD-1+ Treg (middle panel) and CD69+ Treg (right panel) in B16-F10 melanoma expressing (FL) or (Del) HIF-1α. Data are reported as the percentage (%) of live cells for Treg quantification or mean florescence intensity (MFI) for PD-1 and CD69 expression and represent the mean ± SEM (error bars) of 14 mice per group, (each dot represents one mouse). Statistically significant differences (indicated by asterisks) are calculated relative to (FL) tumors using an unpaired two-tailed Student’s t-test (ns= not significant; * = p<0.05).

**(C)** Flow cytometry dot plot analysis of NK cells in representative blood samples from HIF-1α (FL) and HIF-1α (Del) B16-F10 tumor-bearing mice untreated (isotype) or treated with anti-NK1.1 antibody. The NK cell subset was defined in the selected cell population as CD3- NK1.1^+^ cells.

**(D)** Flow cytometry dot plot analysis of CD8 cells in representative blood samples from HIF-1α (FL) and HIF-1α (Del) B16-F10 tumor-bearing mice untreated (Isotype) or treated with anti-CD8a antibody. The CD8^+^ cell subset was defined in the selected cell population as CD3+ CD8+ cells.

**Legends for supplementary Figure 2**

**(A)** RT-qPCR quantification of *Ccl2* and *Ccl5* gene expression in HIF-1α (FL) and HIF-1α (Del) B16-F10 tumors. Data are reported as fold change (FC). Results represent the mean ± SEM (error bars) of 6 mice per group (each dot represents one mouse). ns = not significant, ** =p< 0.005, calculated using an unpaired two-tailed Student’s *t*-test.

**(B)** RT-qPCR quantification of Ccl2 and Ccl5 gene expression in HIF-1α (FL) and HIF-1α (Del) B16-F10 cells cultured under normoxia (21% pO_2_, N) or hypoxia (0.1% pO_2_, H) conditions for 24 hours. Data are reported as fold change (FC). All results are shown as mean ± SEM. ns = not significant, * = p<0.05 calculated using an unpaired two-tailed Student’s *t*-test.

**(C)** XTT assay to evaluate the doubling time of HIF-1α (FL) and HIF-1α (Del) 4T1 cells at indicated time intervals under hypoxia (0.1% p0_2_) conditions. The average of eight replicates is reported. The error bars represent mean ± SEM. ns = not significant determined by an unpaired two-tailed Student’s *t*-test.

**(D)** RT-qPCR quantification of *Ca-9* and *Slc2a1* gene expression in HIF-1α FL (FL) and HIF-1α Del (Del) 4T1 cells cultured under normoxia (21% pO_2_, N) or hypoxia (0.1% pO_2_, H) conditions for 24 hours. Results are reported as fold change (FC) and represent the average of three independent experiments. Error bars indicate ± SEM. *** = p<0.001 determined by unpaired two-tailed Student’s *t*-test.

**(E)** Western-blot analysis of HIF-1α and Glut-1 protein expression in HIF-1α FL and HIF-1α Del 4T1 cells cultured under normoxia (21% pO_2_, N) or hypoxia (0.1% 0_2_ pO_2_, H) conditions for 24 hours. Actin was used as a loading control.

**(F)** ELISA quantification of CCL2 and CCL5 released in the supernatant of HIF-1α FL and HIF-1α Del 4T1 cells cultured under normoxia (21% pO_2_, N) or hypoxia (0.1% pO_2_, H) conditions for 24 hours. Results are reported as mean ± SEM from three independent experiments. ns = not significant, * = p<0.05, **, P < 0.01, *** = p<0.001 determined by unpaired two tailed unpaired Student’s t test.

**(G)** *Upper panel:* Treatment strategy of B16-F10 cells cultured under hypoxia with different concentrations of ACF (left panel). *Middle panels:* RT-qPCR quantification of Glut-1 (*Slc2a1*) and *Vegf* gene expression in B16-F10 cells cultured under normoxia (21% pO_2_, N) or hypoxia (0.1% pO_2_, H) conditions for 24 hours and treated with different concentrations of ACF (5, 10, 20, and 30 μM) for last 6 hours. Data are reported as fold change (FC) and represent the average of three independent experiments. The error bars represent mean ± SEM. ns = not significant, * = p<0.05, ** = p<0.01, and *** = p<0.001 calculated using an unpaired two-tailed Student’s *t*-test. *Lower panels:* RT-qPCR quantification of Ccl2 and Ccl5 gene expression in B16-F10 cells cultured normoxia (21% pO2, N) or hypoxia (0.1% pO2, H) conditions for 24 hours and treated with different concentrations of ACF (5, 10 and 20 μM) for last 6 hours. Data are reported as fold change (FC) and represent the average of three independent experiments ± SEM. ns = not significant, calculated using an unpaired two-tailed Student’s t-test. All results are shown as mean ± SEM. ns = not significant, * = p<0.05, *** = p<0.001, calculated using an unpaired two-tailed Student’s *t*-test.

**Legends for supplementary Figure 3**

**A-C:** Quantification of the activation marker CD69 (left panels) and exhaustion marker PD-1 (right panels) on NK **(A)**; CD4^+^ eff **(B)** and CD8^+^ T cells **(C)** infiltrating control and ACF-treated B16-F10 tumors. Data are reported as the percentage (%) of CD45 cells and represent the mean ± SEM (error bars) of 5 tumors per group (each dot represents one tumor). Statistically significant differences (indicated by asterisks) are calculated compared to control tumors using an unpaired two-tailed Student’s t-test (ns= not significant; * = p<0.05).

**D:** Individual curves of tumor growth in mice treated with isotype (control), anti-PD-1, TRP-2_(180-188)_, ACF, double (anti-PD-1 + TRP-2) and triple (anti-PD-1 + TRP-2 + ACF) combinations.

**Legends for supplementary Table 1**

Information about the TCGA melanoma patient described in Figure 4C. The table shows the TCGA patient and sample IDs; mRNA expression and status of CCL5; survival status and time; Winter hypoxia score and mRNA expression of markers for: NK (NCR1 and NCR3); CD3 (CD3D and CD3E); CD4 and CD8 (CD8A and CD8B).
