# Supplementary figures and images for: Targeting HIF-1 alpha transcriptional activity drives cytotoxic immune effector cells into melanoma and improves combination immunotherapy
Source: Oncogene. 2021 Jun 21;40(28):4725–35. doi: 10.1038/s41388-021-01846-x (PMC8282500; doi:10.1038/s41388-021-01846-x)

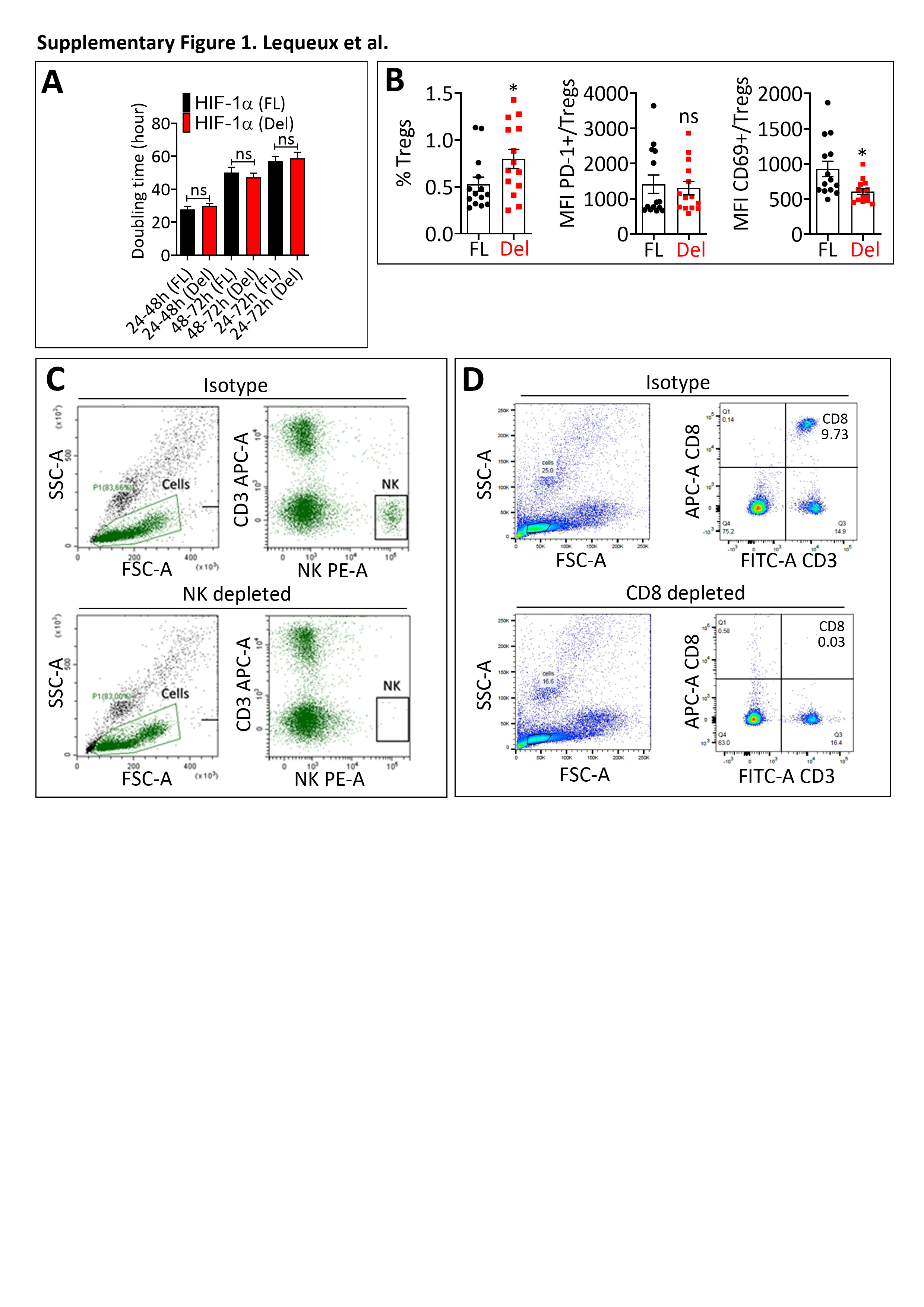

Supplement: Supplementary file 3 — Supp Fig 1 [file 41388_2021_1846_MOESM3_ESM.jpg]

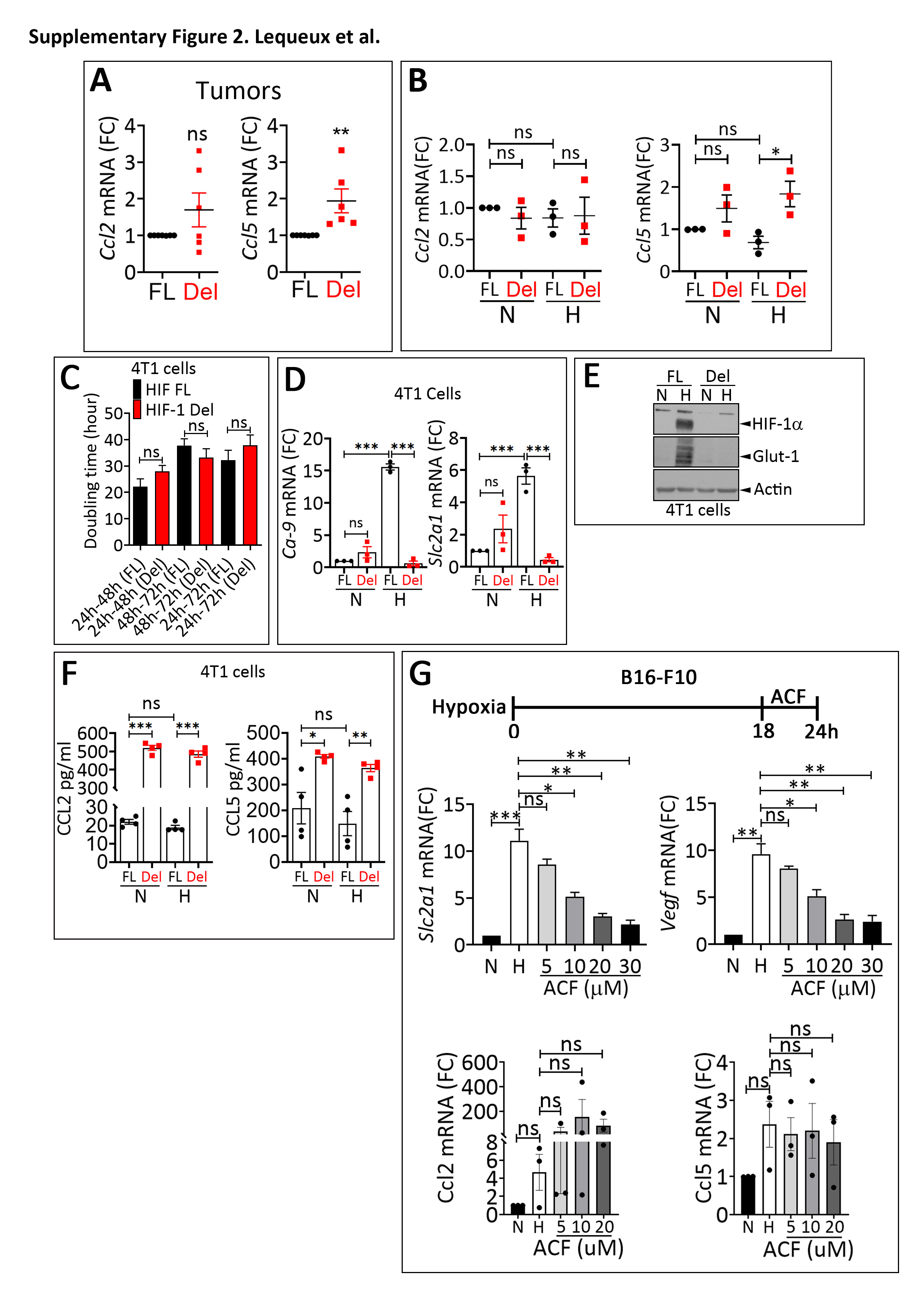

Supplement: Supplementary file 4 — Supp Fig 2 [file 41388_2021_1846_MOESM4_ESM.jpg]

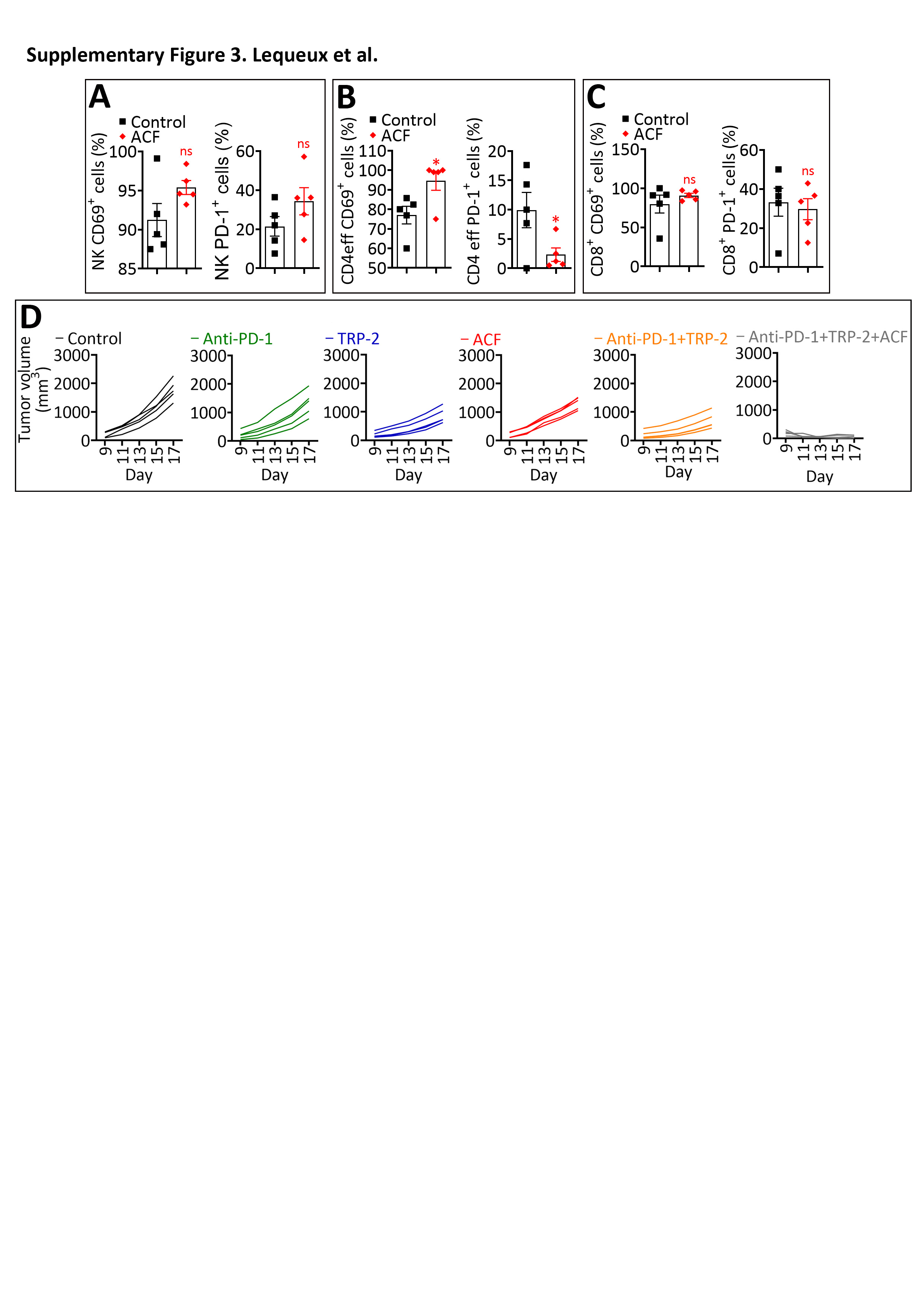

Supplement: Supplementary file 5 — Supp Fig 3 [file 41388_2021_1846_MOESM5_ESM.jpg]
